# Effect of Blood Pressure Variability on Outcomes in Emergency Patients with Intracranial Hemorrhage

**DOI:** 10.5811/westjem.2020.9.48072

**Published:** 2021-01-12

**Authors:** Quincy K. Tran, Daniel Najafali, Laura Tiffany, Safura Tanveer, Brooke Andersen, Michelle Dawson, Rachel Hausladen, Matthew Jackson, Ann Matta, Jordan Mitchell, Christopher Yum, Diane Kuhn

**Affiliations:** * University of Maryland School of Medicine, Department of Emergency Medicine, Baltimore, Maryland; † University of Maryland School of Medicine, The R Adams Cowley Shock Trauma Center, Department of Emergency Medicine, Baltimore, Maryland; ‡ University of Maryland School of Medicine, The Research Associate Program in Emergency Medicine and Critical Care, Department of Emergency Medicine, Baltimore, Maryland; § University of Maryland Medical Center, Department of Neurology, Baltimore, Maryland

## Abstract

**Introduction:**

Patients with spontaneous intracranial hemorrhage (sICH) have high mortality and morbidity, which are associated with blood pressure variability. Additionally, blood pressure variability is associated with acute kidney injury (AKI) in critically ill patients, but its association with sICH patients in emergency departments (ED) is unclear. Our study investigated the association between blood pressure variability in the ED and the risk of developing AKI during sICH patients’ hospital stay.

**Methods:**

We retrospectively analyzed patients with sICH, including those with subarachnoid and intraparenchymal hemorrhage, who were admitted from any ED and who received an external ventricular drain at our academic center. Patients were identified by the International Classification of Diseases, Ninth Revision (ICD-9). Outcomes were the development of AKI, mortality, and being discharged home. We performed multivariable logistic regressions to measure the association of clinical factors and interventions with outcomes.

**Results:**

We analyzed the records of 259 patients: 71 (27%) patients developed AKI, and 59 (23%) patients died. Mean age (± standard deviation [SD]) was 58 (14) years, and 150 (58%) were female. Patients with AKI had significantly higher blood pressure variability than patients without AKI. Each millimeter of mercury increment in one component of blood pressure variability, SD in systolic blood pressure (SBP_SD_), was significantly associated with 2% increased likelihood of developing AKI (odds ratio [OR] 1.02, 95% confidence interval [CI], 1.005–1.03, p = 0.007). Initiating nicardipine infusion in the ED (OR 0.35, 95% CI, 0.15–0.77, p = 0.01) was associated with lower odds of in-hospital mortality. No ED interventions or blood pressure variability components were associated with patients’ likelihood to be discharged home.

**Conclusion:**

Our study suggests that greater SBP_SD_ during patients’ ED stay is associated with higher likelihood of AKI, while starting nicardipine infusion is associated with lower odds of in-hospital mortality. Further studies about interventions and outcomes of patients with sICH in the ED are needed to confirm our observations.

## INTRODUCTION

Patients with spontaneous intracranial hemorrhage (sICH) have high mortality and morbidity rates. The 30-day mortality rate was estimated to be 35–52%.^1^ Blood pressure variability (BPV) is an independent risk factor associated with outcomes among these critically ill patients, especially those suspected to have high intracranial pressure.^2^–^4^ BPV is defined as the average of absolute differences between consecutive blood pressure measurements (successive variations in systolic blood pressure [SBP_SV_]) or variations in SBP during a period of time (standard deviation [SBP_SD_]).^3^

Emergency physicians (EP) are among the first clinicians to manage critically ill patients when they first present. Effective management by EPs has been associated with improved patient outcomes.^5^,^6^ However, little is known about the association between EPs’ management and sICH patients’ outcomes once patients leave the emergency department (ED).

It has been shown that acute kidney injury (AKI) results in negative patient outcomes in critically ill patients, but AKI and outcomes among patients with sICH are not well described.^7^,^8^ Furthermore, patients with sICH are at a high risk for developing AKI due to their existing hypertensive nephropathy, which in turn was associated with worse outcomes in patients with sICH.^9^,^10^ However, the correlation between BPV during patients’ ED stay and AKI has not been investigated. In our study, which included patients with either subarachnoid hemorrhage (SAH) or intraparenchymal hemorrhage (IPH), we aimed to elucidate the association of BPV during sICH patients’ ED stay and the development of AKI during their hospitalization. We also studied how effectively EPs managed these critically ill patients’ blood pressures according to guidelines, and whether EPs’ interventions are associated with our primary outcome of in-hospital AKI and our secondary outcomes of discharge home and mortality. We hypothesized that BPV in the ED would be associated with AKI. Furthermore, EPs’ interventions would also be associated with outcomes in these critically ill patients.

## METHODS

### Study Setting and Patient Selection

After obtaining approval from our institutional review board, we retrospectively studied all adult patients who were admitted to our quaternary academic medical center for management of sICH. Patients who were admitted between January 1, 2011–September 30, 2015 and underwent external ventricular drain (EVD) placement at our medical center were eligible. We identified patients from the electronic health records (EHR) during the study period according to the International Classification of Diseases, Ninth Revision (ICD-9), for sICH: code 430.XX or 431.XX, and procedure code 02.21 for EVD placement.^11^,^12^ Patients with SAH and IPH were included. We excluded patients with insufficient ED records or those with traumatic hemorrhage. We also excluded patients whose source of hemorrhage was secondary to tumor, arteriovenous malformations, ischemic stroke, etc, because these patients have different pathophysiology and outcomes from those with sICH.^13^,^14^

**Population Health Research Capsule**

What do we already know about this issue?

*Critically ill patients, including those with ischemic stroke, are at increased risk to develop acute kidney injury (AKI)*.

What was the research question?

Would blood pressure variability (BPV) in the emergency department (ED) be associated with AKI among spontaneous intracranial hemorrhage (sICH) patients?

What was the major finding of the study?

*In sICH, standard deviation in systolic blood pressure was associated with developing AKI. Nicardipine in the ED was associated with lower odds of in-hospital mortality*.

How does this improve population health?

*Our findings provide evidence for clinicians to avoid BPV in the ED and to use nicardipine early for patients with sICH*.

### Outcome Measures

Our primary outcome was the development of AKI during hospital stay. We used the serum creatinine criteria from the Kidney Disease Improving Global Outcomes scale to identify patients with AKI and their respective stage.^15^ We defined stage 1 AKI as a rise in serum creatinine during hospitalization ≥ 0.3 milligrams per deciliter (mg/dL) or a 1.5-times to 1.9-times increase from level at ED presentation. AKI stage 2 occurred if patients’ serum creatinine increased from 2-times to 2.9-times their serum creatinine level at ED presentation. AKI stage 3 occurred when the serum creatinine level increased to ≥ 3-times the level at ED presentation.^15^ Patients with a history of end-stage renal disease (ESRD) were considered as not having AKI, but were still included in the study.

Our secondary outcomes included the percentage of patients achieving goal SBP at ED departure according to previous guidelines, in-hospital mortality, and being discharged home directly from the hospital.^16^ The American Heart Association/American Stroke Association guidelines recommend that clinicians reduce sICH patients’ SBP to ≤ 160 millimeters of mercury (mm Hg).^16^ We also selected discharge home as an outcome because it has been shown to correlate with good functional independence, compared to those patients who are discharged to a rehabilitation center or nursing home.^17^

### Data Collection and Management

The principal investigator (PI) trained research team members, who were not blinded to the study hypothesis, to extract data. Research team members were trained by sets of 10 patients’ charts until inter-raters’ agreements achieved at least 90% with the PI’s data. Data was extracted into a standardized Microsoft Excel spreadsheet (Microsoft Corp, Redmond, WA). Research team members also extracted data in sections to reduce bias. For example, investigators extracting blood pressure records did not have access to outcome data, and vice versa. Another investigator independently checked 20% of the data to maintain 90% inter-raters’ agreements during the data collection phase. The team met every other month to adjudicate disagreements until the data collection was completed.

We collected ED clinical factors (eg, blood pressure, invasive mechanical ventilation, seizure, etc) from ED paper records that accompanied patients if they were transferred from other hospitals’ EDs. From these ED records, we collected details regarding the managements performed by EPs. Only interventions that were completed were recorded. Interventions that were ordered but not performed were not recorded in our dataset. Patients’ demographic data (eg, age, gender, referring facilities, etc), laboratory values during hospitalization, and dispositions were obtained from our medical center’s EHR.

### Blood Pressure Variability (BPV)

BPV was calculated as previously described.^2^–^4^ Since there is no established standard regarding how frequently ED staff record blood pressures in EDs, we extracted four measurements that were most clinically relevant during patients’ ED stay and available for all patients: at ED triage (SBP_Triage_); at ED departure (SBP_Depart_); the highest one (SBP_Max_); and the lowest one (SBP_Min_), according to their chronological order. The values for SBP_Max_ and SBP_Min_ were not the same as SBP_Triage_ and SBP_Depart_. Appendix 1 depicts BPV graphically using the mean SBP_Triage_, SBP_Max_, SBP_Min_, and SBP_Depart_ for all patients. Additionally, the formulas used to calculate successive variations in systolic blood pressure (SBP_SV_) and standard deviation (SD) in systolic blood pressure (SBP_SD_) are presented.

### Sample Size Calculation

We performed a sample size calculation according to a previous study about BPV in critically ill patients who developed AKI.^19^ Based on this study, we determined that we would need at least 124 patients, or 62 patients with acute AKI and 62 without AKI, to detect a difference of three units of SBP_SD_ with α= 0.05 and power of 80%.

### Data Analysis

We reported descriptive analyses with mean (±SD) or median (interquartile range [IQR]). We analyzed continuous data with a Student’s t-test or Mann-Whitney U test as appropriate. We compared categorical data by Pearson’s chi-square test or Fisher’s exact test as appropriate.

To assess association between BPV, clinical factors during patients’ ED stay, and AKI during their hospital stays, we performed a backward stepwise multivariable logistic regression. Independent variables to be included in the multivariable logistic regression were selected a priori and are shown in Appendix 2. All of these independent variables were included in the backward stepwise logistic regression for the models with all patients and the outcomes of interest (AKI, mortality, and discharge home). To reduce the risk of overfitting the multivariable models, we specified the significance level to remove interaction terms from the model as 0.05, which was stricter than the recommended level of 0.10.^20^ We assessed the goodness-of-fit of our models with the Hosmer-Lemeshow test. Models with *p* > 0.05 were considered a good fit.

Since patients with SAH and IPH have different pathologies and mortality according to their disease severity, we first performed a multivariable analysis for all patients, which contained both SAH and IPH patients without including their disease severity scores. We subsequently performed multivariable analyses for each subgroup and included appropriate disease severity. We used the Hunt and Hess scale for patients with SAH and the Intracerebral Hemorrhage score and Functional Outcome in Patients with Primary Intracerebral Hemorrhage score for patients with IPH.

Independent variables with two-tailed *p* < 0.05 were considered statistically significant. We performed statistical analyses with Minitab version 19 (Minitab LLC, State College, PA) and SigmaPlot version 13 (www.systatsoftware.com, San Jose, CA). The bar graph was generated using GraphPad Prism version 8.3.1 (GraphPad Software, San Diego, CA).

## RESULTS

### Patient Characteristics

**Figure 1:**
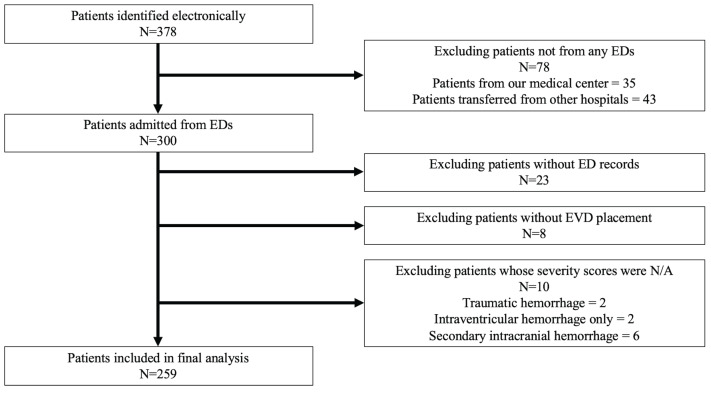
Patient selection diagram with patients included in final analysis. *ED*, emergency department; *EVD*, external ventricular drain; *N/A*, not applicable.

We identified 378 eligible patients electronically and analyzed 259 patients (Figure 1). In our patient population, 71 (27%) developed AKI during hospitalization. There were no patients with ESRD in our patient population. The total number of individuals with AKI also met the requirements of our sample size calculation.

Patients’ mean age (± SD) was 58 (14) years, and 150 (58%) were female (Table 1). The majority of patients (69%) had SAH, while 31% had IPH. The mean intracranial opening pressure (± SD) for our patients was 22 (7) centimeters water. Patients who developed AKI during hospitalization had significantly higher mean serum creatinine levels at ED presentation, when compared to those who did not develop AKI [1.3 (1.3) vs 0.8 (0.5), *p* = 0.002]. The median time interval from admission to the development of AKI in days [IQR] was 23 [3–38] days (Table 1).

### Managements in Emergency Departments

Overall, the median [IQR] of the total number of ED interventions was similar between patients with or without AKI (3 [1–5] vs 3 [2–4], *p* = 0.64) (Table 2). Forty-two (59%) patients with AKI required invasive mechanical ventilation, compared to 101 (54%) patients without AKI. Both groups of patients received similar amounts of intravenous (IV) crystalloids in the ED. The percentage of patients receiving nicardipine infusion was also similar between groups (31% vs 26%, *p* = 0.38).

### Blood Pressure Variability and Patients’ Outcomes

Patients with AKI had significantly higher mean SBP_SD_ than patients without AKI (48 [24] mm Hg vs 38 [20] mm Hg, *p* = 0.002) (Table 1). Similarly, patients with AKI also had higher SBP_SV_ (36 [23] vs 28 [20] mm Hg, *p* = 0.01). While bivariate analyses showed that mortality and hospital length of stay (LOS) were similar between both groups (Table 1), only 8 (11%) patients with AKI were discharged home directly, compared with 49 (26%, *p* = 0.01) patients without AKI.

**Figure 2:**
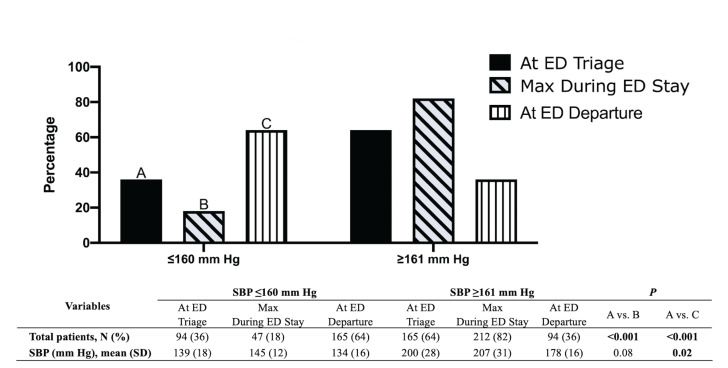
Percentage of patients with systolic blood pressure measurements ≤160 millimeters mercury (mm Hg) or ≥161 mm Hg at different time intervals in the emergency department (ED): at ED triage; during ED stay; and at ED departure. *ED*, emergency department; *mm Hg*, millimeters of mercury; *SD*, standard deviation; *SBP*, systolic blood pressure.

Our study showed that 94 (36%) patients had a SBP at ED triage (SBP_Triage_) of ≤ 160 mm Hg, with 47 (18%) patients having a maximum SBP (SBP_Max_) of ≤ 160 mm Hg during their ED stay (Figure 2). At ED departure, 165 (64%) patients had a SBP (SBP_Depart_) ≤ 160 mm Hg. This increment in percentage of patients whose SBP at ED departure met the recommended guidelines was statistically significant (*p* = <0.001) (Figure 2).

Multivariable logistic regression showed that patients’ increased presenting serum creatinine levels (OR 2.4, 95% confidence interval [CI] 1.4–4.2, *p* = 0.002) and higher SBP_SD_ in EDs (OR 1.02, 95% CI, 1.005–1.03, *p* = 0.007) were significantly associated with increased likelihood of developing AKI during hospitalization (Table 3). Our result suggested that each unit increment of SBP_SD_ in mm Hg is associated with 2% increased likelihood of developing AKI. The goodness-of-fit test with Hosmer-Lemeshow’s *P*-value for this test was > 0.05.

For the secondary outcome of mortality (Table 4), more advanced age was consistently associated with higher odds of in-hospital death in all patients (OR 1.03, 95% CI, 1.005–1.05, *p* = 0.017) and the subgroup of patients with SAH (OR 1.05, 95% CI, 1.004–1.09, *p* = 0.03) (Appendix 3). Each increased year of age was associated with a 3% increased risk of mortality. Similarly, starting nicardipine infusion in EDs was associated with lower odds of death in all patients (OR 0.35, 95% CI, 0.15–0.77, *p* = 0.01) and subarachnoid subgroup (OR 0.19, 95% CI, 0.4–0.82, *p* = 0.027). Additionally, higher Hunt and Hess scale (OR 3.9, 95% CI, 1.5–10.3, *p* = 0.006) and higher ICH score (OR 2.1, 95% CI, 1.2–3.9, *p* = 0.014) were significantly associated with higher odds of death for subarachnoid and intraparenchymal subgroups, respectively (Appendix 3). All three models showed goodness-of-fit tests with *p* > 0.05.

For the secondary outcome of discharge home directly from the hospital (Table 4), increased age was associated with decreased odds of being discharged home in all patients (OR 0.96, 95% CI, 0.94–0.98, *p* = 0.004). Each increased year of age was associated with a 4% lower likelihood of being discharged home. Increased age (OR 0.97, 95%, CI 0.94–0.99, *p* = 0.03) and a higher Hunt and Hess scale (OR 0.51, 95% CI, 0.37–0.71, *p* = 0.001) were associated with lower likelihood of being discharged home among the subgroup of patients with SAH (Appendix 3). Goodness-of-fit tests’ *p*-values for both models were > 0.05. We could not perform subgroup analysis for patients with IPH and discharge home, as there were not enough outcome measures to perform reliable multivariable logistic regression analyses.

## DISCUSSION

Our study demonstrated that a 10-unit mm Hg increase of SBP_SD_ during patients’ ED stay was associated with a 2.7-fold likelihood of developing AKI. Patients’ absolute level of blood pressure, specifically their SBP_Max_ and SBP_Min_, did not have a significant association with AKI. Furthermore, we did not demonstrate an association between BPV with mortality or discharge home. Factors associated with patients’ secondary outcomes were related to their disease severity (eg, age, the need for mechanical ventilation in ED) but nicardipine infusion by EPs was associated with decreased likelihood of patients’ in-hospital mortality.

The association between BPV and AKI has yet to be established for sICH patients. In critically ill patients with stroke, AKI has been associated with worse patient-centered outcomes by negatively impacting discharge disposition and mortality.^21^–^23^ Blood pressure control has been highlighted in stroke patients to contribute to renal injury, but this association remains unclear in the sICH patient population, especially in the hyperacute phase. Moreover, renal insufficiency that goes unrecognized has been shown to be common in stroke patients and negatively impact their short-term outcomes.^24^,^25^ Our study suggests that one component of BPV during patients’ ED stay, the SBP_SD_, was significantly associated with sICH patients developing AKI during their hospital stay.

Patients who experience a sudden elevation of blood pressure, such as those with hypertensive sICH, would experience a condition called pressure natriuresis.^26^ Patients with this condition would experience a significant increase of urinary sodium excretion and volume diuresis causing them to be intravascularly depleted.^26^ As a result, it is recommended that EPs’ monitor these high-risk patients’ volume statuses closely since hypovolemia may precipitously lower patients’ blood pressure. Furthermore, EPs’ should consider using nicardipine infusion early to smoothly reduce patients’ blood pressure toward the goal of a SBP ≤ 160 mm Hg to comply with the American Heart Association’s guidelines.^16^ Nicardipine infusion has been shown to reduce blood pressure toward this goal more rapidly, while producing less BPV than intravenous push (IVP) antihypertensive medication.^27^–^29^

Although our study did not identify BPV as an independent risk factor for the outcome of mortality among patients with sICH, we identified one EPs’ intervention that was associated with patients’ in-hospital mortality. Starting nicardipine infusion in the ED was associated with 65% lower likelihood of death for patients. This observation was present in the all-patient group, and was confirmed in the SAH subgroup, after adjusting for their appropriate disease severity. However, this effect was not present in the IPH subgroup, likely due to its smaller sample size. Nicardipine infusion was shown to produce less BPV compared to other IVP antihypertensive medications (eg, labetalol, hydralazine).^28^,^29^ Therefore, it is possible that patients in our study who received nicardipine infusion in the ED had less BPV and were less likely to develop hematoma expansion as well as neurological deterioration.^18^,^30^,^31^ Further studies are needed to confirm our observations and to further investigate EPs’ interventions and sICH patients’ outcomes.

The percentage of patients who developed AKI in our sICH patient population agreed with a previous study’s result.^10^ From bivariate analyses, without adjusting for any other confounding factors, Ansaritoroghi et al reported that the causes of AKI were attributed to nephrotoxic antibiotics and contrast media.^10^ Therefore, we could not exclude other causality between SBP_SD_ and AKI in our study. Patients who had higher BPV could have been more critically ill and thus may have had to undergo more imaging studies and interventions. These interventions in turn predisposed patients to even higher risks of developing AKI during their hospital stay.

## LIMITATIONS

Our study had several limitations. Due to its retrospective nature, we could not account for different factors affecting patients’ clinical care. Our study also relied on paper ED records, which could have been inadequate in critically ill patients.^32^ We only included four blood pressure measurements, which may have affected the overall values of BPV, as a previous study involving the hyperacute phase of patients with sICH used five blood pressure measurements.^2^ However, a previous report in abstract format reported that the number of blood pressure measurements was not associated with different BPV among patients with ischemic stroke undergoing thrombectomy.^33^ Additionally, a survey of hospitals reported that there is currently no established protocol for the management and frequency of blood pressure measurements in patients with sICH.^34^ Therefore, we extracted blood pressure measurements available for all patients to ensure a more uniform analysis.

Since patients’ baseline creatinine levels were unknown, we used serum creatinine as the criteria to determine AKI. This may have underestimated the incidence of AKI, as many at-risk patients have already developed higher serum creatinine levels when they present to EDs. Since the majority of our patient population was transferred from other hospitals, we could not follow up with them to determine whether AKI developed during hospitalization was permanent or if their creatinine function returned to baseline after discharge. Also, our study only included patients who required EVD after admission, which may limit our study’s generalizability. We were unable to perform subgroup analysis for patients with IPH and the outcome of discharge home because this subgroup did not have enough patients who were discharged home. By using a stricter than usual criteria to enter and remove variables, our stepwise multivariable regressions may have eliminated otherwise eligible independent variables. The design of the Minitab (version 19) statistical software does not retain any information regarding non-significant variables in the stepwise multivariable logistic regressions. As a result, we cannot present the statistical information for these non-significant independent variables from our regressions.

Our study also possessed a few strengths. We demonstrated the association between BPV in the ED with a comorbidity for patients with sICH. Furthermore, we provided evidence that nicardipine infusion in EDs would be associated with lower odds of death for these critically ill patients, although further studies are necessary to confirm our observations.

## CONCLUSION

Our study suggests greater SBP_SD_ during patients’ ED stay is associated with development of AKI during hospital stay among patients with sICH. Furthermore, we identified that starting nicardipine infusion in the ED was significantly associated with a 65% reduction in patients’ odds of in-hospital mortality. Further studies about managements in EDs and outcomes of patients with sICH are needed to confirm our observation.

## Supplementary Information







## Figures and Tables

**Table 1:** Characteristics of patients with spontaneous intracranial hemorrhage who were transferred from emergency departments (ED)to a tertiary care center.

Variables	All patients (N = 259)	AKI during hospital stay (N = 71)	No AKI (N = 188)	P-value†
Age (years), mean (Standad deviation [SD])	58 (14)	57 (13)	59 (14)	0.15
Gender, N (%)				
Male	109 (42)	38 (54)	71 (38)	**0.022**
Female	150 (58)	33 (46)	117 (62)	
Transport type, N (%)				
Ground	175 (68)	48 (68)	127 (68)	0.99
Air	84 (32)	23 (32)	61 (32)	
ESI, median [IQR]^a^	2 [1–3]	2 [1–3]	2 [1–3]	0.80
Ground distance (kilometers), mean (SD)	29 (40)	28 (37)	30 (41)	0.70
ED LOS (minutes), mean (SD)	222 (161)	214 (139)	225 (169)	0.59
Type of hemorrhage, N (%)				
Subarachnoid hemorrhage (SAH)	180 (69)	42 (59)	138 (73)	**0.026**
Intraparenchymal hemorrhage (IPH)	79 (31)	29 (41)	50 (27)	
Disease severity for patients with SAH				
Hunt & Hess scale*, median [IQR]	3 [2–4]	3 [2–4]	3 [2–4]	0.32
Disease severity for patients with IPH				
Intracerebral Hemorrhage score*, mean (SD)	2 (1)	2 (1)	3 (1)	0.84
FUNC score#, mean (SD)	7 (2)	8 (2)	7 (2)	0.55
Seizure, N (%)	27 (10)	10 (14)	17 (9)	0.24
Serum sodium (mEq/L), mean (SD)	141 (5)	141 (4)	141 (5)	0.94
Serum creatinine (mg/dL), mean (SD)	1.0 (0.8)	1.3 (1.3)	0.8 (0.5)	**0.002**
Platelet (count/μL), mean (SD)	235 (79)	225 (81)	239 (79)	0.20
International normalized ratio, mean (SD)	1.1 (0.3)	1.1 (0.3)	1.1 (0.3)	0.48
Serum glucose (mg/dL), mean (SD)	165 (62)	169 (68)	163 (59)	0.56
SBP_Max_ (mm Hg), mean (SD)	196 (37)	211 (41)	190 (35)	**<0.001**
SBP_Min_ (mm Hg), mean (SD)	136 (26)	141 (29)	135 (25)	0.09
SBP_Max-Min_ (mm Hg), mean (SD)	59 (36)	70 (40)	55 (34)	**0.01**
SBP_SV_ (mm Hg), mean (SD)	30 (21)	36 (23)	28 (20)	**0.01**
SBP_SD_ (mm Hg), mean (SD)	41 (21)	48 (24)	38 (20)	**0.002**
ICU SBP (mm Hg), mean (SD)	147 (25)	151 (26)	145 (25)	0.11
ICU admission GCS, median [IQR]	9 [6–14]	9 [7–14]	9 [6–14]	0.66
Intracranial opening pressure (cm H_2_O), mean (SD)	22 (7)	22 (7)	22 (7)	0.76
AKI within 24 hours of admission, N (%)	26 (10)	25 (35)	1 (1)	**<0.001**
Time interval to AKI (days), median [IQR]	N/A	23 [3–38]	N/A	N/A
Any AKI during hospitalization, N (%)				
AKI-level 1	52 (20)	52 (73)	N/A	N/A
AKI-level 2 and 3	19 (7)	19 (27)	N/A	N/A
Mortality, N (%)	59 (23)	21 (30)	38 (20)	0.11
Hospital length of stay (days), median [IQR]	21[14–30]	23 [12–33]	20 [14–28]	0.31
Discharge home, N (%)	57 (22)	8 (11)	49 (26)	**0.01**

* Higher score, more severe disease.

# Higher score, better outcome.

a Emergency Severity Index (ESI) is ranked from 1 (most severe) to 5 (least severe). Patients who were assigned a lower score are associated with higher acuity and higher care intensity in the ED.

† Bold cells indicate statistically significant findings

*AKI*, acute kidney injury; *cm H_2_O*, centimeters of water; *count/μL*, count per microliter; *SBP_Max-Min_*, difference between maximum and minimum systolic blood pressure; *FUNC score*, Functional Outcome in Patients with Primary Intracerebral Hemorrhage score; *GCS*, Glasgow Coma Scale; *ICU*, intensive care unit; *IQR*, interquartile range; *SBP_Max_*, maximum systolic blood pressure; *mEq/L*, milliequivalents per liter; *mg/dL*, milligrams per deciliter; *mm Hg*, millimeters of mercury; *SBP_Min_*, minimum systolic blood pressure; *N/A*, not applicable; *SBP_SD_*, standard deviation in systolic blood pressure; SBP_SV_, successive variations in systolic blood pressure.

**Table 2:** Emergency department management of patients with spontaneous intracranial hemorrhage.

Variables	All patients (N = 259)	AKI during hospital stay (N = 71)	No AKI (N = 188)	P-value
Total number of interventions, median [IQR]	3 [1–4]	3 [1–5]	3 [2–4]	0.64
Total amount of IVF (mL), mean (SD)	258 (549)	266 (647)	238 (485)	0.74
ED mechanical ventilation, N (%)	143 (55)	42 (59)	101 (54)	0.43
Any sedation, N (%)	102 (39)	32 (45)	70 (37)	0.25
Propofol infusion, N (%)	66 (25)	23 (32)	43 (23)	0.12
IVP benzodiazepines, N (%)	43 (17)	15 (21)	28 (15)	0.23
Any paralytics, N (%)	55 (21)	17 (24)	38 (20)	0.51
Succinylcholine, N (%)	23 (9)	6 (8)	17 (9)	0.88
Rocuronium, N (%)	32 (12)	11 (15)	21 (11)	0.35
Any pain medication, N (%)	82 (32)	20 (28)	62 (33)	0.46
Fentanyl infusion, N (%)	5 (2)	1 (1)	4 (2)	0.99
IVP morphine equivalent (unit), mean (SD)	3 (5)	2 (4)	3 (5)	0.11
Any hyperosmolarity therapy, N (%)	41 (16)	13 (18)	28 (15)	0.50
Any antihypertensive medication, N (%)	131 (51)	40 (56)	91 (48)	0.26
IVP labetalol, N (%)	68 (26)	22 (31)	46 (24)	0.29
Nicardipine infusion, N (%)	70 (27)	22 (31)	48 (26)	0.38
Any blood product, N (%)	10 (4)	5 (7)	5 (3)	0.14
Platelet, N (%)	2 (1)	1 (1)	1 (1)	0.47
Fresh frozen plasma, N (%)	8 (3)	4 (6)	4 (2)	0.22
Packed RBCs, N (%)	0 (0)	0 (0)	0 (0)	N/A
Any arterial line monitoring, N (%)	5 (2)	1 (1)	4 (2)	0.99

*AKI*, acute kidney injury; *ED*, emergency department; *IQR*, interquartile range; *IVF*, intravenous fluid; *IVP*, intravenous push; *mL*, milliliter; *N/A*, not applicable; *RBCs*, red blood cells; *SD*, standard deviation.

**Table 3:** Backward stepwise multivariable logistic regression to measure the association between clinical variables and primary outcome: acute kidney injury during hospitalization. All a priori selected variables were included, but only significant variables were reported.

Variables	OR	95% CI	P-value
Serum creatinine – each mg/dL	2.4	1.4–4.2	0.002
SBP_SD_ – each mm Hg	1.02	1.005–1.03	0.007

Hosmer-Lemeshow goodness-of-fit test: degrees of freedom = 8, χ2 value= 15.7, p > 0.05.

*CI*, confidence interval; *mg/dL*, milligrams per deciliter; *mm Hg*, millimeters of mercury; *OR*, odds ratio; *SBP_SD_*, standard deviation in systolic blood pressure.

**Table 4:** Backward stepwise multivariable logistic regressions to measure associations between clinical variables and secondary outcomes: mortality and discharge home. All a priori selected variables were included but only significant variables were reported.

	All patients		
	
	OR	95% CI	P-value
Outcome: mortality			
Age	1.03	1.005–1.05	0.017
ED MV	5.6	2.7–11.6	0.001
Nicardipine infusion	0.35	0.15–0.77	0.01
Outcome: discharge home			
Age	0.96	0.94–0.98	0.004
ED MV	0.2	0.1–0.4	0.001

Mortality and all patients, Hosmer-Lemeshow test: degrees of freedom = 8, χ2= 7.76, P = 0.45.

Discharge home and all patients, Hosmer-Lemeshow test: degrees of freedom = 8, χ2= 10.1, P = 0.26.

*CI*, confidence interval; *ED*, emergency department; *MV*, invasive mechanical ventilation; *OR*, odds ratio.
