# Supplementary material for: Effect of Blood Pressure Variability on Outcomes in Emergency Patients with Intracranial Hemorrhage
Source: West J Emerg Med. 2021 Jan 12;22(2):177–85. doi: 10.5811/westjem.2020.9.48072 (PMC7972364; doi:10.5811/westjem.2020.9.48072)
Supplement: Supplementary file 1 [file wjem-22-177-s001.docx]

**Appendix 1.** Calculation of successive variations in systolic blood pressure (SBP_SV_) and standard deviation in systolic blood pressure (SBP_SD_), as well as a graphical depiction of the four clinically relevant systolic blood pressures extracted: 1) SBP_Triage_ ; 2) SBP_Max_; 3) SBP_Min_ ; and 4) SBP_Depart_. The successive variations are shown with arrows between each SBP and the standard deviation is shown with vertical arrows from the SBP measurement of interest and the overall mean SBP.


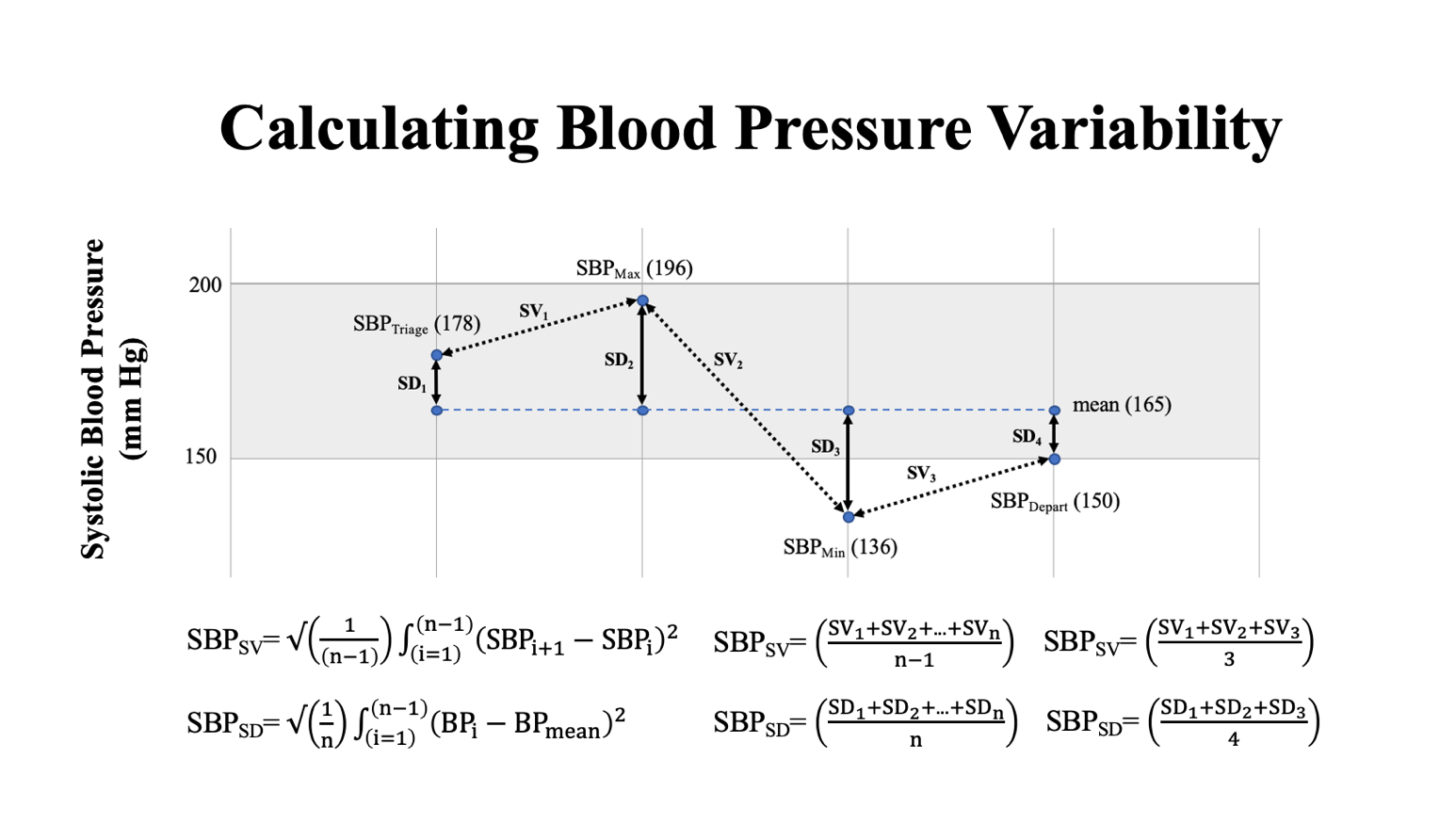
 *BP*, blood pressure; *SBP_Max_*, maximum systolic blood pressure; *mm Hg*, millimeter of mercury; *SBP_Min_*, minimum systolic blood pressure; *SD*, standard deviation; *SBP_SD_*, standard deviation in systolic blood pressure; *SV*, successive variations; *SBP_SV_*, successive variations in systolic blood pressure; *SBP*, systolic blood pressure; *SBP_Depart_*, systolic blood pressure at departure; *SBP_Triage_*, systolic blood pressure at triage.
