# Supplementary material for: Effect of Blood Pressure Variability on Outcomes in Emergency Patients with Intracranial Hemorrhage
Source: West J Emerg Med. 2021 Jan 12;22(2):177–85. doi: 10.5811/westjem.2020.9.48072 (PMC7972364; doi:10.5811/westjem.2020.9.48072)
Supplement: Supplementary file 2 [file wjem-22-177-s002.docx]

**Appendix 2**. Independent continuous and categorical variables used in multivariable logistic regression analyses. All variables were included in the regressions. Only statistically significant factors were reported.

| **Independent variables for logistic regressions** | |
| --- | --- |
| **Continuous variables** | **Categorical variables** |
| Age − each year | Gender − Female= 1, Male= 0 |
| ICH score | Mechanical ventilation − Yes= 1, No= 0 |
| FUNC score | Seizure − Yes= 1, No= 0 |
| Hunt & Hess scale | Any sedation − Yes= 1, No= 0 |
| ESI − each unit | Any paralytics − Yes= 1, No= 0 |
| SBP_Max-Min_ − each mm Hg | Fentanyl infusion − Yes= 1, No= 0 |
| SBP_Max_ – each mm Hg | Any hyperosmolarity therapy − Yes= 1, No= 0 |
| SBP_Min_ – each mm Hg | Infusion nicardipine − Yes= 1, No= 0 |
| SBP_SV_ − each mm Hg | Any IVP − labetalol − Yes= 1, No= 0 |
| SBP_SD_ − each mm Hg | Any blood products − Yes= 1, No= 0 |
| INR − each unit | Type of hemorrhage − IPH= 1, SAH= 0 |
| Sodium − each mEq/L |  |
| Creatinine − each mg/dL |  |
| Platelet − each count |  |
| Glucose − each mg/dL |  |
| Total IVP MEU − each unit |  |
| Volume of IVF received − each mL |  |
| ED LOS − each minute |  |
| **Outcomes** | |
| Primary – AKI |  |
| Secondary − discharge home & mortality |  |

*AKI*, acute kidney injury; *SBP_Max-Min_*, difference between maximum and minimum systolic blood pressure; *ED*, emergency department; *ESI*, emergency severity index; *FUNC score*, Functional Outcome in Patients with Primary Intracerebral Hemorrhage score; *INR*, international normalized ratio; *ICH score*, Intracerebral Hemorrhage score; *IPH*, intraparenchymal hemorrhage; *IVF*, intravenous fluid; *IVP*, intravenous push; *LOS*, length of stay; *SBP_Max_*, maximum systolic blood pressure; *mEq/L*, milliequivalents per liter; *mg/dL*, milligram per deciliter; *mL*, milliliter; *mm Hg*, millimeter of mercury; *SBP_Min_*, minimum systolic blood pressure; *MEU*, morphine equivalent unit; *SBP_SD_*, standard deviation in systolic blood pressure; *SAH*, subarachnoid hemorrhage; *SBP_SV_*, successive variations in systolic blood pressure.
