# Supplementary material for: Effect of Blood Pressure Variability on Outcomes in Emergency Patients with Intracranial Hemorrhage
Source: West J Emerg Med. 2021 Jan 12;22(2):177–85. doi: 10.5811/westjem.2020.9.48072 (PMC7972364; doi:10.5811/westjem.2020.9.48072)
Supplement: Supplementary file 3 [file wjem-22-177-s003.docx]

**Appendix 3.** Backward stepwise multivariable logistic regression to measure associations between clinical variables and secondary outcomes: mortality and discharge home. Multivariable logistic regression for the subarachnoid hemorrhage (SAH) subgroup contained the Hunt & Hess scale, while the regression for intraparenchymal hemorrhage (IPH) contained the Intracerebral Hemorrhage (ICH) score.

|  | **All patients** | | | **SAH patients only** | | | **IPH patients only** | | |
| --- | --- | --- | --- | --- | --- | --- | --- | --- | --- |
|  | **OR** | **95% CI** | ***P*** | **OR** | **95% CI** | ***P*** | **OR** | **95% CI** | ***P*** |
| Outcome: mortality |  |  |  |  |  |  |  |  |  |
| Age | 1.03 | 1.005-1.05 | 0.017 | 1.05 | 1.004-1.09 | 0.03 | NS | NS | NS |
| ED MV | 5.6 | 2.7-11.6 | 0.001 | NS | NS | NS | NS | NS | NS |
| Nicardipine infusion | 0.35 | 0.15-0.77 | 0.01 | 0.19 | 0.4-0.82 | 0.027 | NS | NS | NS |
| Hunt & Hess scale | NA | NA | NA | 3.9 | 1.5-10.3 | 0.006 | NA | NA | NA |
| ICH score | NA | NA | NA | NA | NA | NA | 2.1 | 1.2-3.9 | 0.014 |
| Outcome: discharge home |  |  |  |  |  |  |  |  |  |
| Age | 0.96 | 0.94-0.98 | 0.004 | 0.97 | 0.94-0.99 | 0.03 | NS | NS | NS |
| ED MV | 0.2 | 0.1-0.4 | 0.001 | NA | NA | NA | NS | NS | NS |
| Hunt & Hess scale | NA | NA | NA | 0.51 | 0.37-0.71 | 0.001 | NA | NA | NA |

Mortality and all patients, Hosmer-Lemeshow test: degrees of freedom = 8, χ2= 7.76, p= 0.45

Mortality and SAH patients, Hosmer-Lemeshow test: degrees of freedom = 8, χ2= 15.9, p= 0.43

Mortality and IPH patients, Hosmer-Lemeshow test: degrees of freedom = 8, χ2= 4, p= 0.83

Discharge home and all patients, Hosmer-Lemeshow test: degrees of freedom = 8, χ2= 10.1, p= 0.26

Discharge home and SAH patients, Hosmer-Lemeshow test: degrees of freedom = 8, χ2= 13, p= 0.11

*We did not perform subgroup analysis for patient with IPH because there were not enough patients in this group who were discharged home for reliable analysis.

*CI*, confidence interval; *ED*, emergency department; *ICH score*, intracerebral hemorrhage score; *IPH*, intraparenchymal hemorrhage; *MV*, invasive mechanical ventilation; *NS*, no significant association in multivariable logistic regression; *NA*, not applicable; *OR*, odds ratio; *SAH*, subarachnoid hemorrhage.
